# Effect of Subject Rotation on Assessment of Esthetic Dental Ratios: A Simulation Study

**DOI:** 10.1155/2016/3957806

**Published:** 2016-03-22

**Authors:** Rajesh Gyawali, Prabhat Ranjan Pokharel, Jamal Giri

**Affiliations:** Department of Orthodontics, College of Dental Surgery, B.P. Koirala Institute of Health Sciences, Dharan 56700, Nepal

## Abstract

*Objective*. This study aimed to find out the change in esthetic ratios during rotation of patient's head using a simulation.* Materials and Methods.* A plaster study model was photographed placing its midline along the long axis of the camera. Then a series of photographs were taken by rotating the model each degree till 10° on both right and left sides. These photographs were digitally measured and the ratio of the maxillary anterior teeth at zero-degree rotation was compared with that at various degrees of rotation.* Results*. As the model was rotated to the right side till 10°, the ratio of the right lateral to central incisor gradually decreased while the ratio of the left lateral to central incisor gradually increased. However, the ratio of the canine to lateral incisor on both sides gradually increased. Similar results were obtained when the model was rotated to the left side. The ratio of the lateral to central incisor deviated from the acceptable range (±10%) when there was rotation of more than 7°, whereas the ratio of the canine to lateral incisor was within the acceptable range till 10° rotation on either side.* Conclusions*. Rotation of the model by more than 7° leads to a substantial change in the esthetic ratio.

## 1. Introduction

A beautiful smile adds beauty to the face and, in today's world, everyone wants to look beautiful. The increasing esthetic demands of the patients have led clinicians to think more about the facial and dental esthetics. Although function, esthetics, and stability are equally important from the point of an orthodontist, esthetics receives highest priority among the patients [1]. Hence, an orthodontist or esthetic dentist should have knowledge regarding esthetic assessment of face and smile.

Planning any treatment requires consideration of the elements of the smile design pyramid: psychology, health, function, and esthetics [2]. Although esthetics is subjective and difficult to quantify, various guidelines are established to help the clinician optimize it satisfying other treatment objectives [3]. Sarver classified esthetic analysis of face as macroesthetics, miniesthetics, and microesthetics in four dimensions [4], all of which are equally important in achieving a good smile. As maxillary anterior teeth are the prominent and dominant teeth during smile, the assessment of the proportional relationship between the widths is an essential tool in esthetic planning of a patient. Golden ratio or the golden proportion is the oldest and most widely used geometric proportion suggested as a guide for the evaluation of the esthetic appearance of maxillary anterior teeth [5]. This ratio considers the apparent width of a lateral incisor to be 62% the size of the central, the canine 62% the size of the lateral, and so on. Later Ward suggested the use of recurrent esthetic dental proportion to describe the successive width of the maxillary anterior teeth from the midline [6].

The proportional relationship of maxillary anterior teeth has been described in the literature by measurements made in the cast [7, 8] or patient's photograph [9–13] or photograph of the cast [14]. But the photographic measurement gives a true result only when the long axis of the lens of the camera is along the midsagittal line of the patient during photography. Rotation of the camera and/or patient's head during capturing the photograph gives altered result. Some authors used specially designed stabilizer for head to standardize the photograph [15]; however, no studies were found to assess the effect of rotation of the head in the esthetic ratios. Hence the aim of this study was to find out the change in esthetic ratios during rotation of patient's head using a simulation.

## 2. Materials and Methods

**Figure 1:**
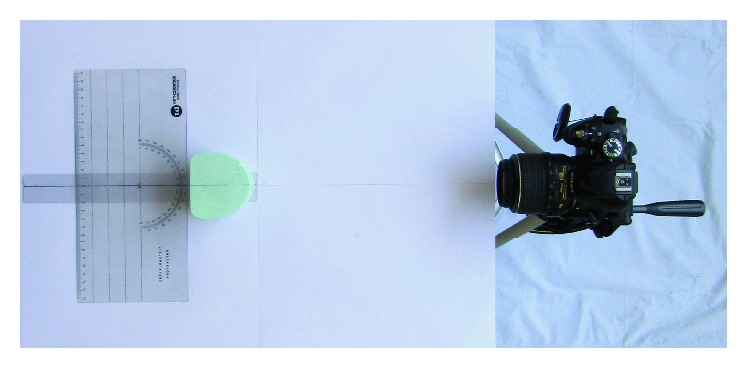
Arrangement of the camera and model.

A plaster study model was taken and placed in the arm of the cephalometric protractor (TD Orthodontics) in such a way that the central line of the arm was along the midline of the model and both of them pointing at 90 degrees. All of these were placed on a table at one end, whereas, at the other end, there was a Nikon D5100 DSLR camera mounted on a tripod. The long axis of the lens was along the central line of the arm of the protractor. This arrangement placed the midline of the study model along the long axis of the lens. The distance between the lens and the study model was kept 60 cm which is almost the same used during intraoral photography (Figure 1).

**Figure 2:**
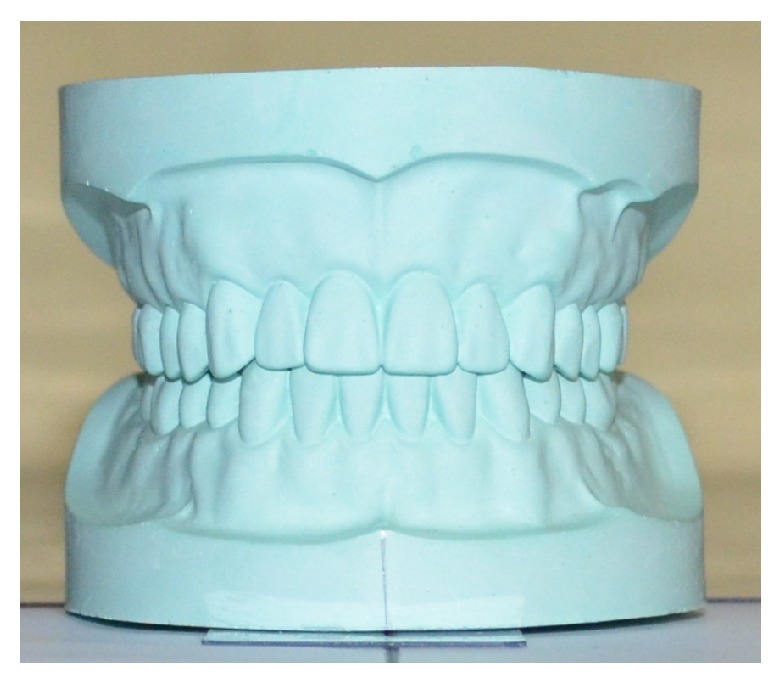
Photograph of the model at zero-degree rotation.

**Figure 3:**
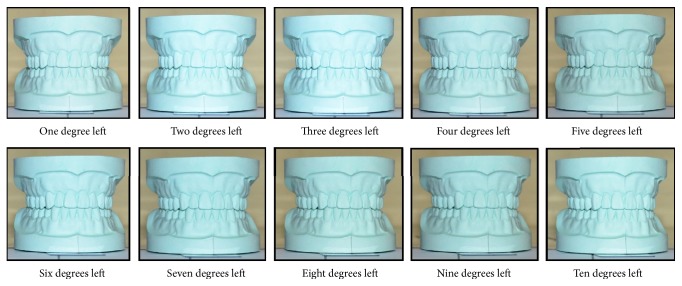
Photograph of the model at various degrees rotation to the left.

**Figure 4:**
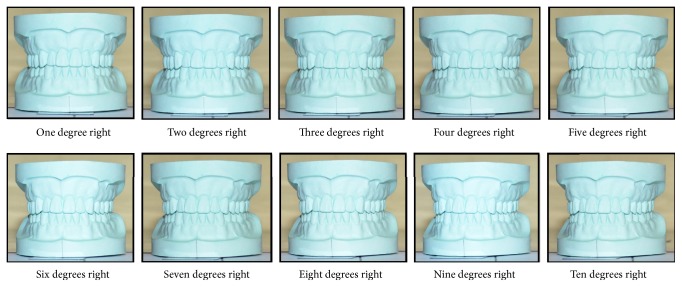
Photograph of the model at various degrees rotation to the right.

With these preparations, the first photograph was taken (Figure 2). A second photograph was taken by rotating the model one degree to the left. It was done by moving the horizontal arm of the protractor. Again, it was rotated by 2 degrees and the photograph was taken. The same process was repeated till 10 degrees (Figure 3) and the similar procedure was done by rotating the model to the right side (Figure 4). All of these photographs were uploaded into a computer and the apparent width of maxillary anterior teeth of the model was measured by software Image J 1.49 (freely available at http://imagej.nih.gov/ij/). The cervicoincisal length of maxillary left central incisor was measured in the model and the photograph for calibration of the digital image in the software.

All the measurements were done three times, each measurement at one week apart by a single investigator. The average of these three measurements was taken to calculate the ratio of maxillary lateral to central and canine to lateral at various angulations. All the data were entered in Microsoft Excel and the ratios at various angulations were compared with that at zero-degree angulation.

## 3. Results

The mean apparent mesiodistal width of maxillary anterior teeth was calculated at zero-degree angulation and at each degree angulation on both sides up to ten degrees (Table 1). The ratio of maxillary lateral to central incisor and canine to lateral incisor was also calculated at all angulations (Table 2).

As the model was rotated to the right side from zero to ten degrees, the ratio of right lateral to central incisor gradually decreased while the ratio of the left lateral to central incisor gradually increased. However, the ratio of the canine to lateral incisor of both sides gradually increased. The similar results were obtained when the model was rotated to the left side (Table 2).

Considering the acceptable deviation of the ratio from the ideal (without rotation) as −10% to + 10% [16], the ratio of lateral to central incisor deviates from the acceptable range when there was rotation of more than 7 degrees on either side (Tables 2 and 3). When the ratio of the canine to lateral incisor was evaluated, it was within the limits of ten percent till the model was rotated up to 10 degrees on either side.

## 4. Discussion

For the esthetic assessment in photographic studies, the photographs were uploaded to the computer and the measurements made using various computer programs. The individual tooth measurements were then made to calculate the ratios of maxillary anterior teeth. The literature shows differing opinions regarding the ideal ratio to be used as a guideline during the esthetic finishing of the case. Some authors advocate golden proportion [5], golden percentage [17], recurring esthetic dental proportion [6], and Preston's Proportion [18] whereas some stressed modification of golden proportion as per the differing races [10, 19–21]. Ignoring the fixed ratio or percentage, evaluation of unique dentofacial presentation and esthetic perception of each individual has also been advised [7, 22, 23]. Photographic techniques in those photographic studies were least focused and the effect of rotation on the esthetic ratios is not discussed in orthodontic literature.

Rotation of patient's head or camera during the photography may affect the apparent size of the maxillary teeth, leading to misinterpretation of the esthetic ratios. Rotation of the head to one side leads to decrease in the apparent dimension of teeth on that side. In the present experiment, rotating the model to the left had led to an increase in the apparent mesiodistal dimension of teeth on right side and vice versa. Hence the esthetic ratios depending upon those dimensions would differ when the model was rotated.

When the model was rotated to the left, the apparent width of the left central incisor, lateral incisor, and canine decreases. The decrease in the dimension of the central incisor was less when compared to the lateral incisor and canine which might be due to the increasing curvature of the arch from central incisor to canine. At the same time, the apparent dimension of right lateral incisor and canine increases but right central incisor decreases. It is due to the reason that left canine and lateral incisor from the curved position of the arch come to the front, perpendicular to the long axis of the camera whereas the central incisor moves away from the long axis of the camera.

Rotating the model to the left decreases the apparent size of the left lateral incisor and canine and the change in the left canine to lateral incisor ratio was slightly less as compared to left lateral to central incisor ratio because the size of central incisor was decreased to a lesser extent. On the other hand, rotating model to the left increases the apparent dimension of right canine and lateral incisor. The increase in the dimension of right canine was more compared to right lateral incisor which leads to increase in the right canine to lateral incisor ratio. The apparent dimension of the right lateral incisor increases, and the central incisor decreases, hence the ratio of right lateral to central incisor also increases as the model was rotated to the left.

During rotation of the model by each degree, change in the ratio of the canine to lateral incisor was less gradual as compared to the ratio of the lateral to central incisor (Table 2). As a result of which lateral to central incisor ratio crosses the acceptable limits of −10% to +10% after seven degrees of rotation, whereas the ratio of the canine to lateral incisor was within the limits till ten degrees of rotation.

This study compared the ratio of the mesiodistal dimension of teeth, not the absolute value. Hence, the results would be similar if the study model with uniformly smaller or larger sized teeth is used. Mesiodistal dimensions of teeth are uniformly larger in crowded case as compared to normal or spaced arch [24]. Therefore, similar results are expected when photographs of patients with different sized teeth are evaluated for esthetic ratios.

## 5. Conclusions

Rotation of the model by more than seven degrees leads to a substantial change in the esthetic ratio. Hence photographic studies to assess the esthetic dental ratios should be undertaken, taking care of the rotation of the camera and/or subject.

Similar studies in patients with larger sample size are recommended to validate the result obtained from this simulation study.

## Figures and Tables

**Table 1:** Apparent dimension of maxillary anterior teeth of the model in centimeter.

Angulation	Right canine	Right lateral incisor	Right central incisor	Left central incisor	Left lateral incisor	Left canine
Zero-degree	0.477	0.490	0.777	0.780	0.520	0.450
1R	0.473	0.480	0.773	0.770	0.527	0.463
2R	0.467	0.470	0.770	0.770	0.533	0.470
3R	0.453	0.457	0.767	0.770	0.543	0.480
4R	0.447	0.450	0.760	0.767	0.547	0.493
5R	0.443	0.440	0.757	0.767	0.553	0.500
6R	0.443	0.440	0.757	0.767	0.557	0.507
7R	0.437	0.430	0.750	0.767	0.560	0.513
8R	0.427	0.420	0.743	0.767	0.577	0.533
9R	0.417	0.410	0.733	0.760	0.580	0.537
10R	0.400	0.393	0.733	0.760	0.580	0.537
1L	0.480	0.497	0.777	0.763	0.500	0.440
2L	0.483	0.500	0.777	0.760	0.490	0.437
3L	0.493	0.510	0.777	0.747	0.477	0.430
4L	0.503	0.513	0.777	0.747	0.477	0.433
5L	0.510	0.513	0.777	0.740	0.467	0.423
6L	0.510	0.513	0.773	0.740	0.467	0.423
7L	0.537	0.527	0.760	0.730	0.460	0.420
8L	0.550	0.533	0.760	0.723	0.430	0.400
9L	0.550	0.533	0.753	0.723	0.413	0.393
10L	0.567	0.543	0.750	0.717	0.403	0.387

1R: one-degree rotation to the right.

1L: one-degree rotation to the left.

**Table 2:** Tooth ratios at various angulations.

Angulation	Right canine to lateral incisor	Right lateral to central incisor	Left lateral to central incisor	Left canine to lateral incisor
Zero-degree	0.973	0.631	0.667	0.865
1R	0.986	0.621	0.684	0.880
2R	0.993	0.610	0.693	0.881
3R	0.993	0.596	0.706	0.883
4R	0.993	0.592	0.713	0.902
5R	1.008	0.581	0.722	0.904
6R	1.008	0.581	0.726	0.910
7R	1.016	0.573	0.730	0.917
8R	1.016	0.565	0.752	0.925
9R	1.016	0.559	0.763	0.925
10R	1.017	0.536	0.763	0.925
1L	0.966	0.639	0.655	0.880
2L	0.967	0.644	0.645	0.891
3L	0.967	0.657	0.638	0.902
4L	0.981	0.661	0.638	0.909
5L	0.994	0.661	0.631	0.907
6L	0.994	0.664	0.631	0.907
7L	1.019	0.693	0.630	0.913
8L	1.031	0.702	0.594	0.930
9L	1.031	0.708	0.571	0.952
10L	1.043	0.724	0.563	0.959

1R: one-degree rotation to the right.

1L: one-degree rotation to the left.

**Table 3:** Acceptable limits of various ratios.

Tooth ratios	Right canine to lateral	Right lateral to central	Left lateral to central	Left canine to lateral
Acceptable limits (−10% to 10%)	0.876 to 1.070	0.568 to 0.694	0.600 to 0.733	0.779 to 0.952
